# Extensive, Multifocal Cutaneous Leishmaniasis Presenting with Varied Morphologies in an Immunosuppressed Patient

**DOI:** 10.4269/ajtmh.18-0998

**Published:** 2019-08

**Authors:** Ian T. Watson, Ravi C. Patel, Richard R. Jahan-Tigh

**Affiliations:** 1 Texas A&M College of Medicine, Bryan, Texas;; 2 Department of Dermatology, University of Texas Medical School at Houston, Houston, Texas

**Figure 1.**
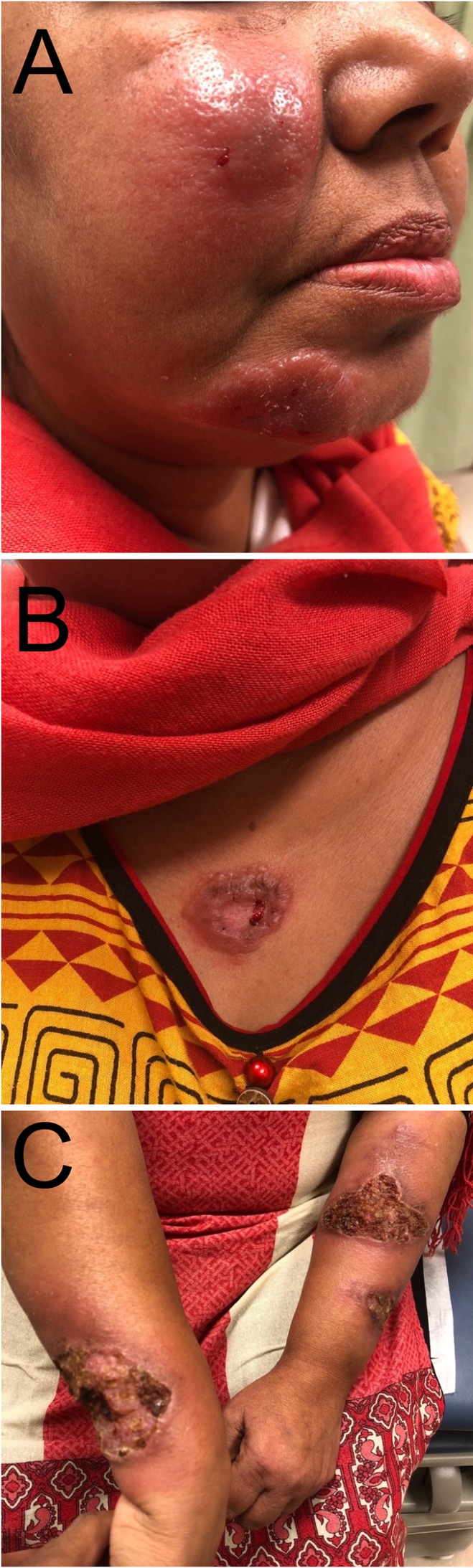
Photograph depicting erythematous, indurated plaques on the face (**A**) and ulcerated, crusted plaques with indurated borders on the upper chest (**B**) and bilateral forearms (**C**). This figure appears in color at www.ajtmh.org.

**Figure 2.**
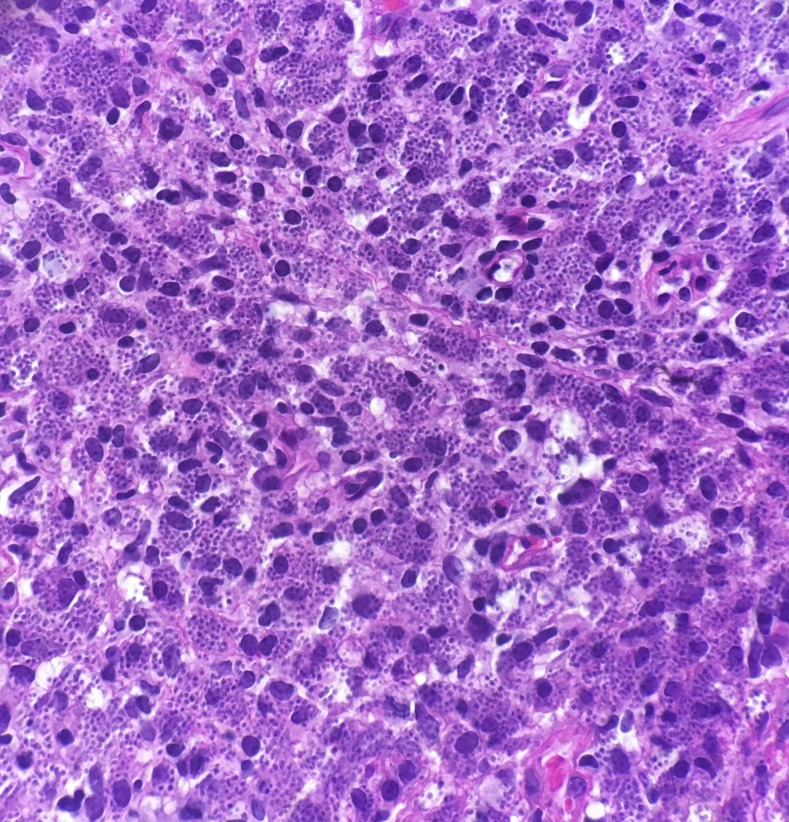
Microscopic histopathologic image demonstrating amastigotes within dermis. Hematoxylin–eosin staining. This figure appears in color at www.ajtmh.org.

**Figure 3.**
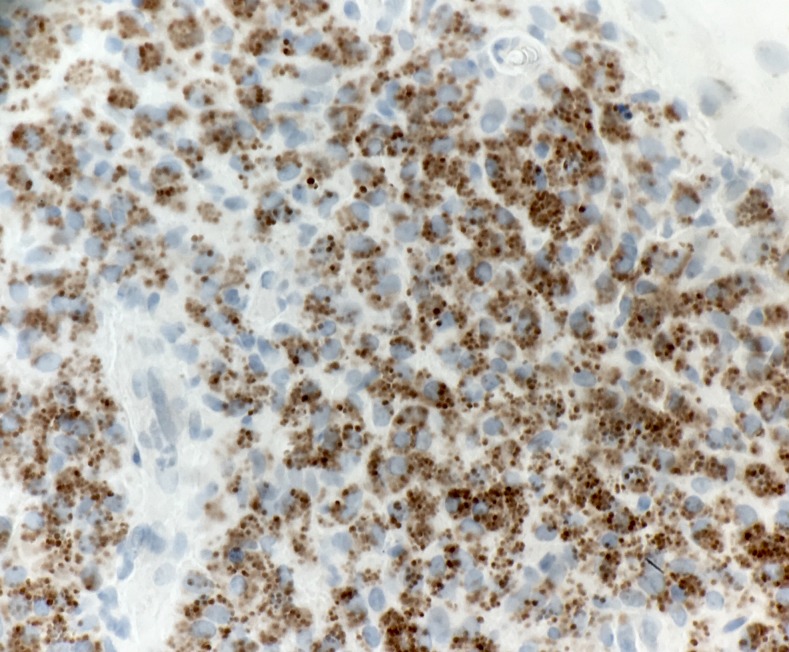
CD1a stain showing dermal parasitized histiocytes with amastigotes. This figure appears in color at www.ajtmh.org.

A 46-year-old woman from Pakistan presented to a clinic in Houston, Texas, with a 10-month history of plaques spread on the face, chest, and bilateral forearms (Figure 1A–C). The patient described progressive enlargement of lesions and associated tenderness. Skin lesions initially started while the patient was a refugee in Turkey. She was given a diagnosis of sarcoidosis at an outside hospital, and was being treated with 15 mg methotrexate weekly and 10 mg prednisone daily at the time of presentation to our clinic. The patient denied fevers, chills, and weight loss. On physical examination, there were erythematous, indurated plaques on the face. The upper chest and bilateral forearms were notable for ulcerated, crusted plaques with indurated borders. Multiple subcutaneous nodules were palpable on the left arm. Clinically, facial lesions were concerning for leprosy. Hemoglobin was 10.6 g/dL, mean corpuscular volume was 66.5 fL/cell, angiotensin converting enzyme was 17 nmol/mL/minutes, and erythrocyte sedimentation rate was 23 mm/hour. Three additional skin biopsies were obtained from left and right forearms and sent for histopathological examination (Figure 2). Histological survey demonstrated numerous intracellular amastigotes in all three biopsies. Staining for CD1a was positive (Figure 3) and was most closely associated with Old World species, which was supported by her travel history. A sample was sent for polymerase chain reaction and followed by DNA sequencing analysis, which showed *Leishmania tropica*. The patient’s disease was refractory to intravenous liposomal amphotericin B, and a course of sodium stibogluconate was complicated by pancreatitis and acute interstitial nephritis leading to cessation of therapy. The patient’s recovery from complications is ongoing, and therapy with miltefosine may be considered in the future.

Leishmaniasis is a tropical infection caused by more than 20 species of protozoa belonging to the genus *Leishmania*. Infection is endemic to Southeast Asia, East Africa, Latin America, and the Mediterranean region and is transmitted by the female phlebotomine sand fly of the *Phlebotomus* and *Lutzomyia* genera.^1^ An annual incidence of 1.2 million most commonly affects children and young adults, and is a leading cause of mortality from parasitic infections globally.^2,3^ Cutaneous manifestations of leishmaniasis are typically distributed to the exposed skin of the face, neck, and extremities.^4^ Clinical suspicion should be raised in patients from endemic regions who present with chronic characteristic skin lesions. Definitive diagnosis of cutaneous and mucocutaneous leishmaniasis is confirmed histologically.^1^ Most patients can be treated topically or intralesionally, and antimonials are still considered a first-line therapy. Systemic therapies, including amphotericin B, miltefosine, antimonials, and azole antifungals, are indicated in patients with multiple or large lesions, involvement of the hands, feet, or face, or with a history of immunosuppression.^5^
